# Seasonal variations in *Plasmodium falciparum* genetic diversity and multiplicity of infection in asymptomatic children living in southern Ghana

**DOI:** 10.1186/s12879-018-3350-z

**Published:** 2018-08-29

**Authors:** Joshua Adjah, Bless Fiadzoe, Ruth Ayanful-Torgby, Linda E. Amoah

**Affiliations:** Department of Immunology, Noguchi Memorial Institute for Medical Research, University of Ghana, Accra, Ghana

**Keywords:** Asymptomatic, Malaria, Allele, *msp* 1, *msp* 2, Genetic diversity, Multiplicity of infection

## Abstract

**Background:**

Genetic diversity in *Plasmodium falciparum* (*P. falciparum)* parasites is a major hurdle to the control of malaria. This study monitored changes in the genetic diversity and the multiplicity of *P. falciparum* parasite infection in asymptomatic children living in southern Ghana at 3 month intervals between April 2015 and January 2016.

**Methods:**

Filter paper blood spots (DBS) were collected quarterly from children living in Obom, a community with perennial malaria transmission and Abura, a community with seasonal malaria transmission. Genomic DNA was extracted from the DBS and used in polymerase chain reaction (PCR)-based genotyping of the *merozoite surface protein* 1 (*msp* 1) and *merozoite surface protein* 2 (*msp* 2) genes.

**Results:**

Out of a total of 787 samples that were collected from the two study sites, 59.2% (466/787) tested positive for *P. falciparum*. The *msp* 1 and *msp* 2 genes were successfully amplified from 73.8% (344/466) and 82.5% (385/466) of the *P. falciparum* positive samples respectively. The geometric mean MOI in Abura ranged between 1.17 (95% CI: 1.08–1.28) and 1.48 (95% CI: 1.36–1.60) and was significantly lower (*p* < 0.01, Dunn’s multiple comparison test) than that determined in Obom, where the geometric mean MOI ranged between 1.82 (95% CI: 1.58–2.08) and 2.50 (95% CI: 2.33–2.678) over the study period. Whilst the *msp* 1 R033:MAD20:KI allelic family ratio was dynamic, the *msp* 2 3D7:FC27 allelic family ratio remained relatively stable across the changing seasons in both sites.

**Conclusions:**

This study shows that seasonal variations in parasite diversity in these communities can be better estimated by *msp* 1 rather than *msp* 2 due to the constantly changing relative intra allelic frequencies observed in *msp* 1 and the fact that the dominance of any *msp* 2 allele was dependent on the transmission setting but not on the season as opposed to the dominance of any *msp* 1 allele, which was dependent on both the season and the transmission setting.

**Electronic supplementary material:**

The online version of this article (10.1186/s12879-018-3350-z) contains supplementary material, which is available to authorized users.

## Background

The high prevalence of malaria in Ghana has resulted in a majority of adults and children older than 5 years attaining a semi-immune status due to past and continuous exposure to different parasite clones [1]. Repeated exposure to a particular parasite clone is said to offer protection from clinical episodes of malaria caused by that parasite clone, which may result in asymptomatic carriage of that clone by the host [2, 3]. The multiplicity of infection (MOI), which identifies the number of clones within a particular infection can serve as a measure of the level of malaria transmission as well as identify hotspots within very low transmission settings [4, 5]. Within-host parasite diversity at the population level and the dynamics of diversity are important in malaria eradication efforts [6, 7]. Malaria parasite diversity is distinct in different individuals, populations, transmission settings and seasons within endemic zones and changes with variations in parasite prevalence [5], and has been suggested to be constantly changing [8–11]. Parasite genetic diversity and MOI studies have also been found to be important in the surveillance of strains circulating in a particular transmission area. The identification of hotspots are important in understanding the epidemiology of *P. falciparum* infections for informed interventions to be instituted [5, 12, 13]. There have been reports of extensive genetic diversity of *P. falciparum* in high transmission areas with some infected people harboring multiple clones of the parasites. On the contrary, people living in low transmission areas tend to have low genetic diversity with a high proportion being monoclonal infections [14–17]. Transmission intensity and efficiency vary significantly between malaria endemic areas and is dependent on several factors including varying mosquito populations, changes in parasite-vector interactions due to temperature changes, hosts’ immunological changes induced by parasite interaction and the spatial heterogeneity over which these occur [18, 19]. Thus, understanding the population and within-host diversity of the malaria parasite in distinct geographic locations with varying malaria transmission patterns across changing malaria seasons, may help identify control measures that are appropriate for a particular transmission setting at a specific time of the year [20].

The most common and widely used tool for the estimation of parasite diversity is PCR based genotyping of a gene with high diversity such as *merozoite surface protein* 1 (*msp* 1*,* PF3D7_0930300), *merozoite surface protein* 2 (*msp 2,* (PF3D7_0206800) and the *glutamate-rich protein* (*glurp*, PF3D7_1035300) [21–25]. The use of PCR to derive the number of repeat length variants observed at the highly diverse loci of the *msp* 1 and *msp* 2 genes helps to measure MOI and transmission in a population. Although only a limited number of loci can be examined at a time and some minor parasite populations missed by conventional PCR [5, 26] compared to more sensitive tools including SNP typing, conventional PCR followed by agarose gel electrophoresis remains an easier, quicker and a more cost effective way to adequately genotype different parasite clones [27]. Many recent studies on parasite diversity, especially in poorly resourced settings that cannot afford to use the enhanced genotyping tools, which are relatively more expensive continue to utilize *msp* genotyping followed by agarose gel electrophoresis as their parasite diversity analysis tool [28–31].

The genetic diversity of *P. falciparum* circulating in the middle belt of Ghana has been extensively characterized [32, 33]. However, diversity studies on parasites circulating in southern Ghana have been limited to selected time points in asymptomatic [27, 31] or in symptomatic infections [34, 35]. This study sought to identify changes in infecting parasite MOI and genetic diversity in asymptomatic children living in different malaria transmission settings over the course of changing malaria transmission seasons. This data would help in the selection and design of appropriate intervention tools that can be deployed during the different transmission seasons.

## Methods

### Study site

**Fig. 1:**
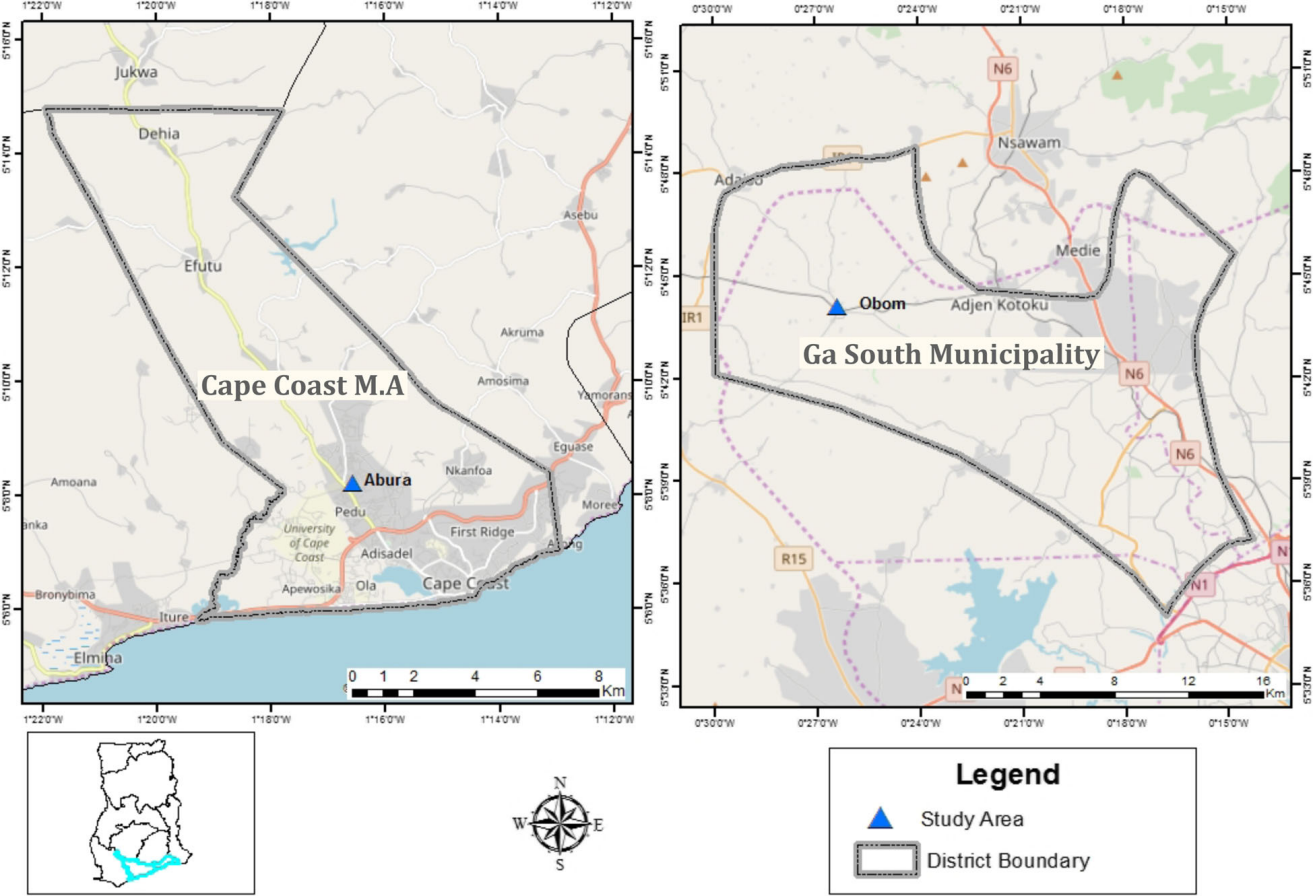
Map of the study sites. A Map of Ghana zooming into the Cape Coast Metropolitan Assembly (CCMA) of the Central Region on the Left and the Ga South Municipality in the Greater Accra Region on the Right

The study was conducted in community primary schools in two areas of southern Ghana; Obom, a semi-rural community in the Ga South Municipality of the Greater Accra Region with high and perennial malaria transmission and Abura, a semi-urban community in the Cape Coast Municipality of the Central Region with low and seasonal malaria transmission [36]. In 2010, the Ghana Statistical Services identified higher levels of overcrowding in sleeping rooms as well as a higher prevalence of liquid waste disposal in the compounds and streets than into drains and gutters in the Ga South municipality than in the Cape Coast Metropolis [37, 38]. The Cape Coast Metropolis also has fewer illiterate inhabitants than the Ga South District [37, 38]. Both study sites lie in the coastal savannah belt of Ghana (Fig. 1) and have most infections occuring in the major rainy season that peaks between June and August. *Plasmodium falciparum* parasite prevalence in Obom has been noted to be higher than in Abura [36, 39].

### Sample collection

After obtaining parental consent, 237 children between the ages of 6 and 12 years were recruited in a multiple cross sectional study; 83 from Obom and 154 from Abura. Filter paper (Whatman® 3 mm) blood spots (DBS) were prepared from ~ 50 μL of finger pricked blood that was collected in April, June and October 2015 as well as in January 2016. The filter paper blots were air-dried and stored desiccated at room temperature. None of the children sampled exhibited any signs or symptoms of clinical malaria at the time of blood draw and were classified as either healthy with no PCR detectable parasites or asymptomatic, harboring PCR detectable *P. falciparum* parasites. The same children were followed up during subsequent sampling visits.

### Extraction of parasite DNA

Total genomic DNA (gDNA) was extracted from the dried blood spot (DBS) using the Chelex extraction procedure [40, 41]. Briefly, two 3 mm discs were punched out from the dried blood spots into 1.5 mL microcentrifuge tubes containing 1 mL of 1X phosphate buffered saline (PBS). The discs were washed twice in 1 mL PBS and then boiled at 99 °C in 200 μL of 20% Chelex (Sigma-Aldrich, USA) in DNase/RNase-free water. After a final centrifugation step (14,000 x *g* for 1 min), the extracted DNA was transferred into a labelled 0.6 mL microcentrifuge tube and then stored at − 20 °C.

### Molecular detection of *P. falciparum* parasites

A nested PCR amplification protocol based on amplification of the small subunit ribosomal RNA (18S rRNA) gene was used to detect *P. falciparum* [42]. The 15 μL primary reaction mixture contained 5 μL of DNA (~ 10–50 ng), 2.5 mM MgCl_2_, 200 nM dNTP mix, 200 nM rPLU5 and rPLU6 primer set (Additional file 1) and 1 U OneTaq DNA polymerase (NEB, UK). The reaction cycling parameters comprised an initial 2 min denaturation at 94 °C followed by 35 cycles of 94 °C for 30 s, 54 °C for 1 min and 68 °C for 1 min, with a final 5 min extension at 68 °C. The secondary reaction mixture was similar to the primary; however, 2 μL of the primary PCR product was used as DNA template and with rFal1 and rFal2 primer set (Additional file 1) used in the amplification. The cycling profile for the secondary PCR was similar to the primary PCR except for the annealing temperature which was increased from 54 °C to 59 °C. A no template negative control and MRA-102G (3D7) and MRA-155G (HB3) positive controls were included in each set of PCR amplifications.

### Allelic genotyping of *msp* 1 *and msp* 2 *genes*

The polymorphic regions of *msp* 1 block 2 and *msp* 2 block 3 were amplified using a protocol adapted from the WHO protocol for malaria parasite genotyping [43] and similar to that described by Ayanful-Torgby et al. [39, 44]. All PCR reactions were carried out in a total volume of 15 μL, containing 200 nM dNTP mix, 2 mM MgCl_2_, 200 nM each of forward and reverse primers for both *msp* 1 and *msp* 2 (M1-OF, M1-OR, M2-OF, M2-OR, Additional file 1) and 0.75 units of OneTaq Polymerase. Four microliters of extracted DNA (~ 10–50 ng) and 1 μL of control genomic DNA (10 ng/mL) was used as template for the primary PCR reaction. The 15 μL secondary PCR reactions contained a similar recipe as the primary reaction except that the template was 2 μL of the primary PCR product and the primers used were M1-KF & M1-KR, M1-MF & M1-MR, RO33-F & RO33-R, S1fw & N5rev and S1fw & M5rev (Additional file 1: Table S1). A no template negative control and positive controls for each allelic family (MRA-102G (3D7) for *msp 2* 3D7 alleles, MRA-159G (KI) for *msp 1* KI and *msp 2* FC27 alleles, MRA-155G (HB3) for *msp 1* MAD20/*msp 2* FC27 alleles and MRA-200G (R033) for *msp 1* RO33/*msp 2* 3D7 alleles) were included in each set of PCR reactions. PCR products were resolved for an average of 50 min at 120 V on a 2% agarose gel stained with 0.5 μg/mL ethidium bromide. After the electrophoresis, the gels were visualized under UV trans-illumination using a Toyobo FAS-III gel doc system and then analyzed.

### Data analysis

A sample containing a multiclonal infection produced more than one amplified fragment after either the three *msp* 1 or the two *msp* 2 allelic family PCR reactions. A sample containing a clonal infection produced a single product after the three *msp* 1 PCR reactions as well as a single product after the two *msp* 2 reactions. A simplified method of estimating multiplicity of infection (MOI), where MOI of a sample was taken as the highest number of PCR products (amplicons) obtained after the family specific *msp* 1 or *msp* 2 PCR amplifications was used. Multiplicity of infection was generalized and reported as the geometric mean MOI for infections in the group of children from one study site at each time point.

The geometric mean, column statistics, unpaired T test and One-way analysis of variance were obtained using GraphPad Prism v5.0. The distribution of the different allelic families was presented as proportions. The frequency of *msp* 1 and *msp* 2 family alleles was calculated as the ratio of the number of PCR products obtained for each family to the total number of gene-specific PCR products identified. The MOI for *msp* 1 or *msp* 2 in a sample was estimated as the total number of distinct *msp* 1 or *msp* 2 PCR amplicons detected in that sample, the total MOI of a sample is the highest number of PCR fragments obtained after both *msp* 1 and *msp* 2 genotyping [39, 45]. A chi-square test as well a a Fishers exact test was performed using R3.3.2-win to determine possible relationships between the occurrence of any *msp* 1 and *msp* 2 allele at the different time points. Statistical significance was defined as *p* value ≤ 0.05 unless otherwise stated.

## Results

At enrolment, there were 46.5 and 45.3% male children in Obom and Abura respectively. The mean (SEM) age of the children in Obom was 8.96 years, which was not significantly different (*p* = 0.8*,* Unpaired T test) from the mean age of 9.10 years in Abura (Table 1). A number of children were lost to follow up mainly because the academic year ended in July and some of the children did not return back to school at the beginning on the new school year, thus the reduced numbers available during the October and January sampling (Additional file 2). Out of a total of 787 samples that were collected from the two study sites, 59.2% (466/787) tested positive for *P. falciparum* (asymptomatic at the time of sampling). Asymptomatic parasite carriage over the course of the study was 41% (197/481), ranging between 13.7% (13/95) and 64% (89/139) in Abura and 88%, ranging between 80% (64/80) and 96.4% (80/83) in Obom (Additional file 2, Fig. 2) and was significantly higher at all time points in Obom than Abura (Mann-Whitney test; U = 1400, *p* < 0.0001 at all time points).

**Table 1:** Demographic data of participants involved in the study from the two sites

	Obom	Abura	*P* value
Age-range (years)	6–12	6–12	
Mean age ± SD	8.96 ± 0.17	9.10 ± 0.12	0.8
Geographic setting	semi-rural	semi-urban	
Average sex ratio (M:F)	1:1	1:1	
MOI^a^ (GM)	2.1	1.4	< 0.0001
MOI (range)	1.82–2.50	1.17–1.48	
N	83	154	

*MOI* Multiplicity of Infection, *GM* Geometric mean (GM); Age range of children enrolled, *SD* Standard deviation, *M/F* Male to Female ratio, *N* initial number of children enrolled

^a^average of the four time points

**Fig. 2:**
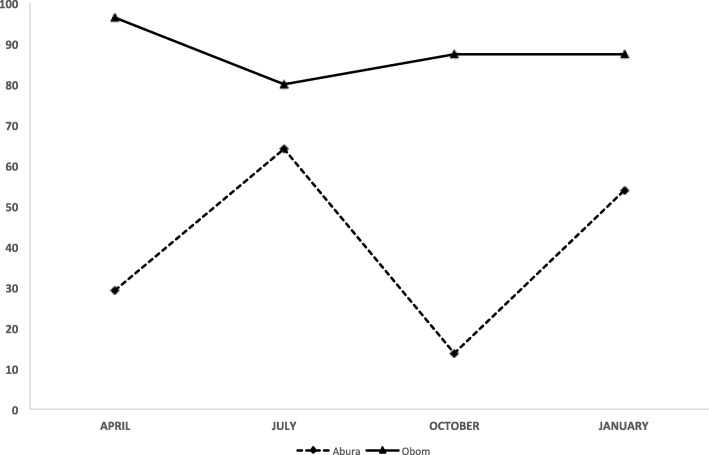
Prevalence of asymptomatic children. Comparison of asymptomatic parasite carriage in Abura (broken line), a low malaria transmission setting and Obom (solid line), a high malaria transmission setting in April, July, October and January (which represent the different malaria transmission seasons pre peak, peak, end of peak and off peak respectively)

### Dynamics of parasite multiplicity of infection

**Fig. 3:**
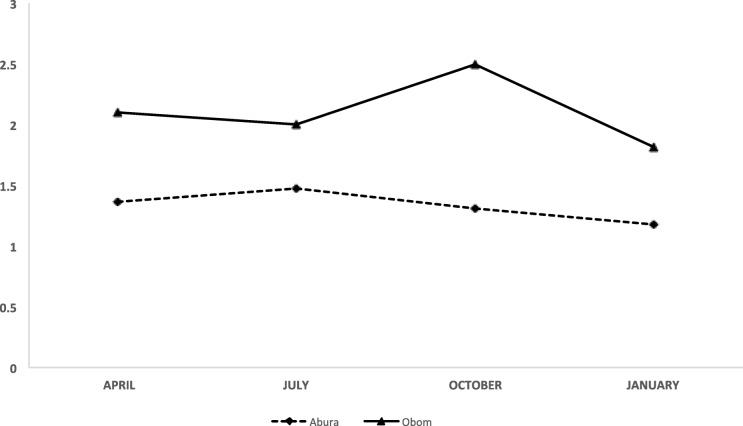
Seasonal multiplicity of infection (MOI). Comparison of the geometric mean MOI (95% confidence interval) among children under 12 years in Abura (broken line), a low malaria transmission setting and Obom (solid line), a high malaria transmission setting in April, July, October and January (which represent the different malaria transmission seasons pre peak, peak, end of peak and off peak respectively)

The highest number of fragments obtained after either the *msp* 1 or *msp* 2 gene amplifications was considered as the number of independent parasite clones co-existing within an infection and as the multiplicity of that infection. The *msp* 1 and *msp* 2 genes were successfully amplified from 73.8% (344/466) and 82.5% (385/466) of the *P. falciparum* positive samples respectively. The samples yielded amplicons compared to the 57.4% of asymptomatic samples that yielded products in Abura. The *msp* 2 amplification efficiencies were similar in both sites, 83% (223/269) in Obom and 82.2% (162/197) in Abura (Additional file 2). Amplification efficiencies for *msp* 1 was higher in Obom where 86% of the asymptomatic samples yielded amplicons compared to the 57.4% of asymptomatic samples that yielded products in Abura. The *msp* 2 amplification efficiencies were similar inboth sites, 83% (223/269) in Obom and 82.2% (162/197) in Abura (Additional file 2). The geometric mean multiplicity of infection (MOI) was however estimated for the entire group of children from each of the study sites. The highest geometric mean MOI of 2.50 (95% CI 2.33–2.68) in children from Obom occurred in October 2015 and was significantly higher (*p* < 0.01, Dunn’s Multiple Comparison test) than the lowest geometric mean MOI of 1.82 (95% CI 1.58–2.08) at the same site, which occurred in January 2016 (Fig. 3). In Abura, the highest geometric mean MOI of 1.48 (95% CI 1.36–1.60), which occurred in July 2015 was not significantly different from the lowest, 1.17 (95% CI 1.08–1.28) in January 2016 (*p* > 0.05, Dunn’s Multiple Comparison test) (Fig. 3). The geometric mean MOI in asymptomatic children from Obom was always significantly higher than those in children from Abura during each visit (*p* < 0.001 at each time point, Mann Whitney Test).

### Dynamics of parasite genetic diversity

The presence of an amplified product after a family-specific PCR was used to categorize the parasites contained in a sample as belonging to that particular allelic family. A number of children were infected with multiple parasite strains and yielded multiple PCR products that belonged to the same as well as different *msp* 1 (RO33, MAD20 and K1) and *msp* 2 (3D7 and FC27) allelic families. Each of the multiple *msp* 1/*msp* 2 PCR products was considered as a different parasite. The *msp* 1 and *msp* 2 alleles were characterized based on the size of the PCR products using 50 bp and 100 bp DNA ladders as size standards. Variations in the sizes of the largest and smallest PCR products within each family specific PCR was noted both *msp* 1 and *msp* 2 family members in the two study sites. The approximate size of the smallest and largest alleles of *msp* 1 was 120 bp and 350 bp respectively for parasites from Obom and 150 bp and 320 bp respectively for parasites from Abura. No obvious trends were observed in the diversity of the smallest and largest sized PCR fragments obtained after *msp* 1 genotyping in both study sites across the various time points. However, in Obom the largest sized *msp* 2 fragment, the 400 bp variant persisted from April through to January while in Abura, no trend in the diversity of the largest and smallest sized *msp* 2 PCR fragments were observed across the time points (Table 2).

**Table 2:** Frequencies of *variant msp 1* and *msp 2* alleles

	APRIL			JULY			OCTOBER			JANUARY		
	*N*	SR (bp)	A (Freq/T)	*N*	SR (bp)	A (Freq/T)	*N*	SR (bp)	A (Freq/T)	*N*	SR (bp)	A (Freq/T)
ABURA												
RO33(68)	3	150–300	250 (8/13)	3	200–230	220 (47/50)	0	ND	ND	1	250	250 (3/3)
MAD20(34)	2	150–250	150 (3/4)	3	200–220	220 (2/4)	3	150–200	150(1/3);	3	200–250	200 (19/24)
									170(1/3);			
									200(1/3)			
K1(53)	1	200	200 (5/5)	9	150–300	250 (24/35)	0	ND	ND	6	200–320	250 (4/10)
3D7(148)	7	200–600	300 (8/26)	10	200–400	280 (21/70)	4	200–400	300 (4/8)	6	250–410	250 (23/33)
FC27(71)	7	250–550	250 (4/13)	10	200–450	300 (8/30)	3	300–500	400 (7/10)	6	300–420	350 (5/14)
OBOM												
RO33(152)	4	200–300	250 (29/33)	3	220–270	220 (47/50)	3	200–250	220 (35/40)	3	200–250	220 (13/29)
MAD20(154)	4	150–250	200 (9/19)	7	180–280	220 (26/65)	3	150–220	150 (15/45);	6	150–280	200 (12/28)
									200 (15/45);			
									220(15/45)			
K1(196)	1	200	200 (33/33)	8	120–270	200 (8/37); 250 (8/37)	12	150–300	270 (25/81)	7	200–300	250 (22/44)
3D7(192)	9	150–400	300 (18/46)	9	250–400	300 (16/56)	8	250–400	350 (30/61)	5	250–400	300 (13/29)
FC27(194)	9	200–350	350 (17/50)	8	350–520	450 (17/49)	10	300–580	400 (15/55)	6	300–500	350 (11/38); 400 (11/38)

*N* total number of different -sized fragments (alleles), representing intra allelic diversity, *SR* range of fragment sizes in bp, *A* the most dominant fragment with its frequency of occurrence (Freq) in brackets, *ND* none detected, *(T)* total number of fragments detected for the allelic family. A number of samples were infected with mixed infections and contained either parasites belonging to more than one allelic family, multiple variants of the same allelic family or a mixture of both

**Fig. 4:**
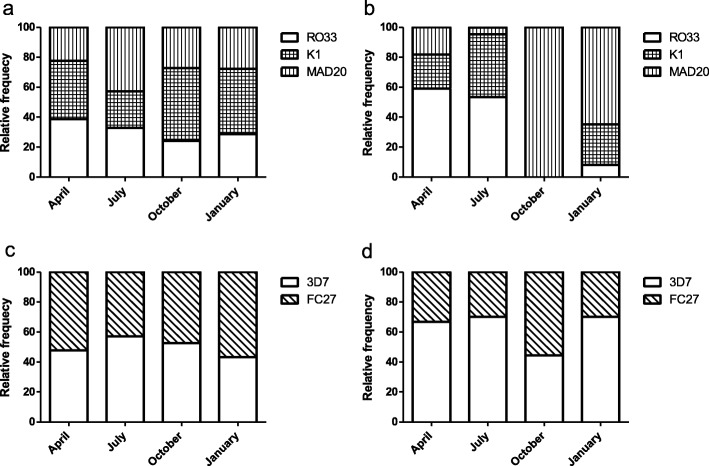
Relative frequency of merozoite surface protein (*msp*) 1 and 2. The percentage of parasites belonging to the different *msp 1* (RO33, MAD20 or K1) and *msp 2* (3D7 or FC27) families relative to the total percentage of parasites belonging to either *msp* 1 or *msp* 2 allelic families in Obom (**a** and **c**) and Abura (**b** and **d**) genotyped across the various time points

In Obom, the proportion of parasites belonging to the R033 of *msp* 1 reduced from 38.8 to 24.1% from April to October 2015 and increased slightly in January 2016 (28.7%). The proportion of parasites belonging to the K1 family reduced from 38.8% in April 2015 to 24.3% in July 2015, increased again in October 2015 to 48.8% and reduced slightly to 43.7% in January 2016. The parasites belonging to MAD20 allelic family increased steadily from April (22.4%) to July 2015 (42.8%), reduced to 27.1% in October 2015 and 27.7% in January 2016 (Fig. 4a). The trend was different than observed in Abura (Fig. 4b). Where, the proportion of parasites with the RO33 was 59.1% in April 2015 with a complete absence in October 2015 and a slight increase of 8.1% in January 2016. The proportion of parasites belonging to the K1 family increased from 22.7% in April 2015 to 40.7% in July 2015 and an absence in October 2015 before increasing again to 27% in January 2016 being the highest. The proportion of parasites belonging to the MAD20 family decreased from 18.2% in April 2015 to 4.7% in July 2015, shot up to 100% in October 2015 and reduced to 64.9% in January 2016 (Fig. 4b). A significant association was identified between the prevalence of any particular *msp* 1 allele and the seasons (time points) in both Obom (χ^2^ = 27.619, df = 6, *p = 0.0001*) and Abura (Fisher’s Exact test, *P = 0.000*) (Fig. 4a & b). Thus the null hypothesis that there was no significant association between the prevalence of a particular *msp* 1 or *msp* 2 allele and a particular season was rejected.

In Obom, the proportion of parasites belonging to the 3D7 *msp* 2 family ranged from 57% in July 2015 to 43.3% in January 2016, while the proportion of parasites belonging to the FC27 family ranged from 43% in July to 56.7% in January 2016 with slight variation between the seasons (Fig. 4c). In Abura, on the other hand, parasites belonging to the 3D7 family increased from 44.4% in October 2015 to 70.2% in January 2016 while the proportion of parasites belonging to the FC27 family decreased from 55.6% in October 2015 to 29.8% in January 2016 (Fig. 4d). No association was identified between the seasons (time points) and the prevalence of a particular *msp* 2 allele in either Obom (χ^2^ = 3.7272, df = 3, *P = 0.2924*) or Abura (Fisher’s Exact test, *P = 0.2057*) (Fig. 4b, d). Thus the null hypothesis that there was no significant association between the prevalence of a particular *msp* 1 or *msp* 2 allele and a particular season was maintained.

## Discussion

This study sought to characterize the genetic diversity of *P. falciparum* and the multiplicity of *P. falciparum* infections over changing malaria seasons in one community with perennial and high malaria transmission and another with seasonal and low malaria transmission. The most prevalent circulating parasite clones at different time points in different transmission settings were also identified.

In Abura a lot children were lost to follow up at between July and October 2015 (Additional file 2), likely as a result of a number of children changing or dropping out of school at the end of the academic year, which runs from September through to July. Other than in October 2015, where the prevalence of asymptomatic infections in the two sites was similar, the prevalence of asymptomatic infections in Obom was significantly higher than asymptomatic infections in Abura. Asymptomatic parasite carriage in Abura was generally low, except for during the peak season (July 2015) when the prevalence was highest. These results support the positive correlation between rainfall and asymptomatic infections, which has been identified in both high and low malaria transmission settings [36, 37] and also the fact that malaria in Abura is low and seasonal. July 2015 registered the lowest proportion of asymptomatic infections in Obom, but was not significantly different from the other time points (*p* > 0.05, Dunn’s Multiple comparison test). This is to be expected in perennial transmission settings, where mosquito vectors and malaria transmission only vary slightly between seasons [46].

The amplification efficiencies for both *msp* 1 and *msp* 2 were lower in Abura compared to Obom most likely because the gDNA extracted from the DBS contained fewer parasites due to lower parasite densities identified in samples from Abura compared to Obom [47]. Generally, PCR amplification efficiencies for single copy genes including *msp* 1 and *msp* 2 are more greatly reduced in the presence of low template concentration than high copy number genes, including the 18 s rRNA gene [48]. The geometric mean MOI for infections in Abura at each time point was significantly lower than that determined in Obom (Fig. 3), most likely because the frequency of infectious mosquito bites in high transmission settings, that could result in additional inoculum of variant parasites is higher than in lower transmission settings [49]. This finding supports general notion that Low transmission settings are commonly associated with reduced MOI in malaria endemic countries [50, 51]. The geometric mean MOI differed significantly across the time points in Abura (Fig. 3), most likely because the mosquito vector population varied between those time points as is expected for communities in semi-urban settings [52]. The low geometric mean MOI identified for Abura, the low transmission setting is consistent with a previous report from Ghana where the MOI recorded in areas with lower malaria transmission were found to be lower than high transmission areas especially during the peak season [53]. The low MOI during the peak season in Obom (Fig. 3) could be as a result of an artifact caused by the presence of a single dominant clone overshadowing the amplification of low-density minor parasite isolates as has been reported in Senegal where the most abundant *msp* 1 and 2 alleles interfered with the amplification of the less abundant one [54, 55]. A recent report from Central Ghana found the wet season (peak malaria season) to be associated with a lower MOI than the preceding dry season [28]. A similarly low geometric mean MOI was identified in Abura (Fig. 3).

There was a wide range of variability in the relative frequencies of different *msp* 1 alleles (inter allelic diversity) as well as the dominance of any *msp*1 allele across the seasons and between the two transmission settings (Table 2). Parasites circulating in both Obom and Abura were predominantly RO33 in April, but K1 in Obom (Fig. 4a) and MAD20 in Abura in October. The K1 allele has similarly been reported to be prevalent in high transmission settings amongst asymptomatic children [38] [46]. The distribution of parasites with different 3D7 and FC27 alleles of *msp* 2 was similar over the course of the study in both Obom and Abura. The approximate 1:1 ratio of 3D7:FC27 *msp* 2 alleles circulating in Obom across the different seasons (Fig. 4c) seems to be a characteristic of parasites circulating in high transmission settings as a similar ratio was observed in Kintampo, a high transmission setting in Ghana between 2009 and 2010 [28]. The 3D7:FC27 ratio of 3:2 in Abura was similarly constant across the different seasons, except for in October (Fig. 4d) where parasite prevalence was extremely low (13.7%).

The variations in *msp* fragment size has been suggested to be the result of a number of genetic events including insertion-deletion mutations and recombination events [56]. The sizes of the *msp* 1 and *msp* 2 alleles ranged from 120 bp to 350 bp and 150 bp to 600 bp respectively (Table 2) and were similar to that previously reported from Gezira State in Central Sudan and in Kolla-Shele area in Southwest Ethiopia where the band sizes for the *msp* 1 and *msp* 2 alleles ranged from 150 to 300 bp for KI, 100–300 bp for RO33, 100–380 bp for MAD20, 250–750 bp for 3D7 and 200–700 bp for FC27 [56–58]. An earlier study conducted in Kintampo in central Ghana identified *msp* 2 alleles with as high as 900 bp [28], which were not encountered in this study. This suggests that different *P. falciparum* parasites circulate in central and southern Ghana, which is not surprising as parasite diversity has been suggested to be constantly changing and distinct in different individuals, populations and seasons [10, 59].

More elaborate studies across different transmission settings are needed to better understand parasite complexity in Ghana.

## Limitations

The resolution of agarose gel electrophoresis does not permit distinction between PCR fragments that differ by a few base pairs, which can result in fewer alleles being detected and consequently, a lower reported MOI. The parasite diversity and MOI recorded in this study could be common to all parasitemic children in the selected communities as no symptomatic children were enrolled in this study for comparison.

## Conclusions

This study shows that seasonal variations in parasite diversity can be better estimated by *msp* 1 genotyping rather than *msp* 2 due to the constantly changing relative intra allelic frequencies observed in *msp* 1 and fact that the dominance of any *msp* 2 allele was dependent on the transmission setting but not on the season as opposed to the dominance of any *msp* 1 allele, which was dependent on both the season and the transmission setting.

## Additional files

Additional file 1: Primers used for *P. falciparum* detection and genotying. (DOCX 17 kb)

Additional file 2: *Plasmodium falciparum* parasite carriage in study participants. (DOCX 15 kb)

## Abbreviations

DBS: Dried blood spot
gDNA: Genomic DNA
MOI: Multiplicity of infection
*msp*: Merozoite surface protein
PBS: Phosphate buffered saline
