# Supplementary material for: Seasonal variations in Plasmodium falciparum genetic diversity and multiplicity of infection in asymptomatic children living in southern Ghana
Source: BMC Infect Dis. 2018 Aug 29;18:432. doi: 10.1186/s12879-018-3350-z (PMC6114730; doi:10.1186/s12879-018-3350-z)
Supplement: Supplementary file 1 — Primers used for P. falciparum detection and genotying. (DOCX 17 kb) [file 12879_2018_3350_MOESM1_ESM.docx]

**Additional file 1: Primers used for *P. falciparum* detection and genotyping**

| **Gene/**  **Primer name** | **Primer sequence** | **Annealing temp** |
| --- | --- | --- |
| ***msp* 1** |  |  |
| M1- OF: | CTAGAAGCTTTAGAAGATGCAGTATTG | 54 °C |
| M1- OR: | CTTAAATAGTATTCTAATTCAAGTGGATCA | 54 °C |
| M1- KF: | AAATGAAGAAGAAATTACTACAAAAGGTGC | 59 °C |
| M1- KR: | GCTTGCATCAGCTGGAGGGCTTGCACCAGA | 59 °C |
| M1- MF: | AAATGAAGGAACAAGTGGAACAGCTGTTAC | 59 °C |
| M1- MR: | ATCTGAAGGATTTGTACGTCTTGAATTACC | 59 °C |
| RO33- F: | TAAAGGATGGAGCAAATACTCAAGTTGTTG | 59 °C |
| RO33- R: | CAAGTAATTTTGAACTCATGTTTTAAATCAGCGTA | 59 °C |
| ***msp* 2** |  |  |
| M2- OF: | ATGAAGGTAATTAAAACATTGTCTATTATA | 54 °C |
| M2- OR: | CTTTGTTACCATCGGTACATTCTT | 54 °C |
| S1fw: | GCTTATAATATGAGTATAAGGAGAA | 50 °C |
| 3D7 N5rev: | CTGAAGAGGTACTGGTAGA | 50 °C |
| FC27 M5rev: | GCATTGCCAGAACTTGAA | 50 °C |
| ***P. f 18S rRNA*** |  |  |
| rPLU6 | TTA AAA TTG TTG CAG TTA AAA CG | 54 °C |
| rPLU5 | CCT GTT GTT GCC TTA AAC TTC | 59 °C |
| rFAL1 | TTA AAC TGG TTT GGG AAA ACC AAA TAT ATT | 59 °C |
| rFAL2 | ACA CAA TGA ACT CAA TCA TGA CTA CCC GTC | 64°C |
