# Supplementary material for: Seasonal variations in Plasmodium falciparum genetic diversity and multiplicity of infection in asymptomatic children living in southern Ghana
Source: BMC Infect Dis. 2018 Aug 29;18:432. doi: 10.1186/s12879-018-3350-z (PMC6114730; doi:10.1186/s12879-018-3350-z)
Supplement: Supplementary file 2 — Plasmodium falciparum parasite carriage in study participants. (DOCX 15 kb) [file 12879_2018_3350_MOESM2_ESM.docx]

**Additional file 2:** *Plasmodium falciparum* parasite carriage in study participants

|  | **April**  **2015** | **July**  **2015** | **October 2015** | **January 2016** | **Total** |
| --- | --- | --- | --- | --- | --- |
| **Obom** |  |  |  |  |  |
| N | 83 | 80 | 79 | 64 | 306 |
| *P. fal* (%N) | 80 (96.4) | 64 (80.0) | 69 (87.3) | 56 (87.5) | 269 (87.9) |
| *msp 1*  *(%P. fal)* | 44 (55.0) | 71 (88.8) | 68 (98.6) | 48 (85.7) | 231 (85.9) |
| *msp 2 (%P.fal)* | 48 (60) | 63 (98.4) | 67 (97.1) | 45 (80.4) | 223 (82.9) |
| **Abura** |  |  |  |  |  |
| N | 154 | 139 | 95 | 93 | 481 |
| *P. fal* (%N) | 45 (29.2) | 89 (64.0) | 13 (13.7) | 50 (53.8) | 197 (41.0) |
| *msp 1*  *(%P. fal)* | 18 (40.0) | 59 (66.3) | 3 (23.1) | 33 (66.0) | 113 (57.4) |
| *msp 2*  *(%P. fal)* | 27 (60.0) | 84 (94.4) | 12 (92.3) | 39 (78.0) | 162 (82.2) |

N, number of children present at sampling; %N, value as a percent (%) of the total number N; *P. fal*, number of children positive for *P. falciparum* parasites after *P. falciparum* 18S rRNA PCR; %*P. fal*, value as a percent (%) of the total number of children positive for *P. falciparum* parasites; *msp* 1, number of samples that produced amplicons after *msp* 1 family specific PCR; *msp* 2, number of samples that produced amplicons after *msp* 2 family specific PCR.
